# Extracellular Vesicles to Predict Outcomes After Transcatheter Aortic Valve Implantation – a Prospective, Multicenter Cohort Study

**DOI:** 10.1007/s12265-024-10521-x

**Published:** 2024-05-28

**Authors:** Radosław Wilimski, Jan Budzianowski, Michał Łomiak, Anna Olasińska-Wiśniewska, Katarzyna Pieniak, Szymon Jędrzejczyk, Olaf Domaszk, Magdalena Chudzik, Krzysztof J. Filipiak, Jarosław Hiczkiewicz, Wojciech Faron, Tomasz Urbanowicz, Marek Jemielity, Marek Grygier, Marcin Grabowski, Mariusz Kuśmierczyk, Bartosz Rymuza, Zenon Huczek, Janusz Kochman, Edwin van der Pol, Rienk Nieuwland, Aleksandra Gąsecka

**Affiliations:** 1 Department of Cardiac Surgery, Medical University of Warsaw, Warsaw, Poland; 2 Club 30”, Polish Cardiac Society, Warsaw, Poland; 3 Department of Interventional Cardiology and Cardiac Surgery, University of Zielona Góra, Collegium Medicum, 65-046 Zielona Góra, Poland; 4 Department of Cardiology, Nowa Sól Multidisciplinary Hospital, 67-100 Nowa Sól, Poland; 5 1St Chair and Department of Cardiology, Medical University of Warsaw, Warsaw, Poland; 6 Department of Cardiac Surgery and Transplantology, Poznan University of Medical Sciences, Poznan, Poland; 7 Department of Hypertensiology, Angiology and Internal Medicine, Poznan University of Medical Sciences, Poznan, Poland; 8 Department of Clinical Sciences, Maria Sklodowska-Curie Medical Academy, Warsaw, Poland; 9 Chair and 1st Department of Cardiology, Poznań University of Medical Sciences, Poznań, Poland; 10 Department of Biomedical Engineering and Physics, Amsterdam UMC, Amsterdam, The Netherlands; 11 Laboratory of Experimental Clinical Chemistry & Amsterdam Vesicle Center, Amsterdam UMC, Amsterdam, The Netherlands

**Keywords:** Aortic stenosis, Extracellular vesicles (EVs), Prognosis, Transcatheter aortic valve implantation (TAVI)

## Abstract

**Introduction:**

Transcatheter aortic valve implantation (TAVI) is an established treatment for aortic stenosis (AS) in patients at intermediate and high surgical risk. Circulating extracellular vesicles (EVs) are nanoparticles involved in cardiovascular diseases. We aimed to (i) determine the effect of TAVI on plasma concentrations of five EV subtypes and (ii) evaluate the predictive value of EVs for post-TAVI outcomes.

**Methods:**

Blood samples were collected 1 day before TAVI and at hospital discharge. Concentrations of EVs were evaluated using flow cytometry.

**Results:**

Concentration of leukocytes EVs decreased after TAVI, compared to the measurement before (p = 0.008). Among 123 patients discharged from the hospital, 19.5% experienced MACCE during the median of 10.3 months. Increased pre-TAVI concentration of phosphatidylserine-exposing EVs was an independent predictor of MACCE in multivariable analysis (OR 5.313, 95% CI 1.164–24.258, p = 0.031).

**Conclusions:**

Patients with increased pre-TAVI concentration of procoagulant, PS-exposing EVs have over fivefold higher odds of adverse outcomes.

**Graphical Abstract:**

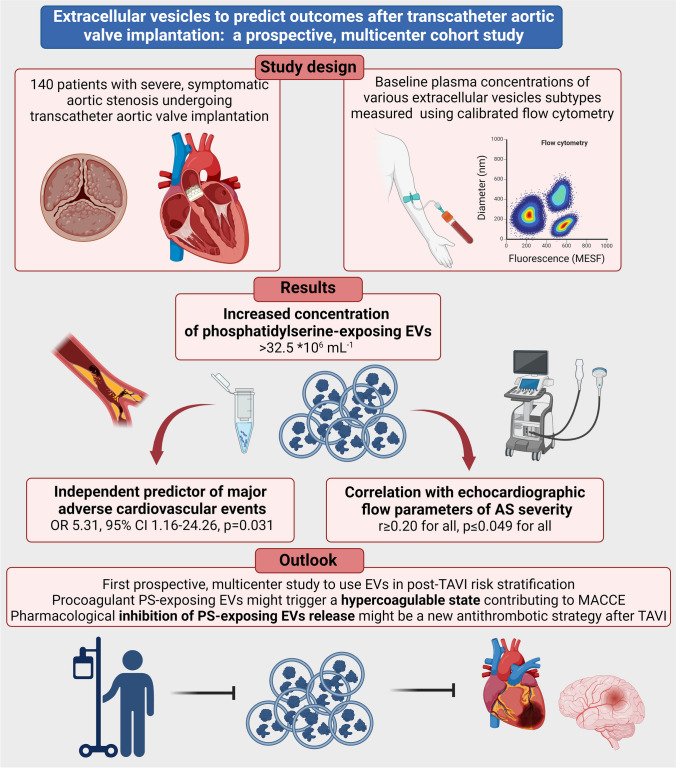

**Supplementary Information:**

The online version contains supplementary material available at 10.1007/s12265-024-10521-x.

## Introduction

Aortic stenosis (AS) is the most common valve disease requiring interventional treatment, with the prevalence increasing with age. AS affects less than 1% of patients in their fifties and almost 10% of octogenarians (1, 2). Progressive degeneration of the aortic valve is a result of fibrous remodeling triggered by complex factors including lipoprotein deposition or chronic inflammation [3]. Clinically, severe AS obstructs blood outflow from the left ventricle, with 50% mortality within 2-years from the moment when the first symptoms occur [4]. The recommended interventions in patients with AS treatment include surgical aortic valve replacement (SAVR) or transcatheter aortic valve implantation (TAVI).

Introduction of TAVI led to a paradigm shift towards minimally invasive procedure and has revolutionized clinical outcomes in AS, especially in inoperable patients [5]. Despite recent advancements in TAVI techniques and careful selection of patients undergoing this procedure, over 15% of patients experience major adverse cardiac and cerebrovascular events (MACCE) in the first year after the intervention [6, 7]. Many factors associated with post-TAVI MACCE have been identified so far, including diabetes or pre-operative anemia, but have not been applied in clinical practice as reliable risk predictors [8, 9]. The most common risk scale for TAVI patients, which is the Society of Thoracic Surgeons (STS) scale underestimates the risk in long-term perspective, especially among high-risk patients [10]. Importantly, STS does not include significant clinical factors that may affect long-term outcomes after TAVI, such as oncologic disease or frailty status. Introduction of novel prediction models to accurately assess risk of post-TAVI MACCE is therefore crucial for the adequate management of post-TAVI.

Extracellular vesicles (EVs) are nanoparticles released from blood cells and vascular endothelium, which are involved in the development of cardiovascular events, including myocardial infarction, stroke, or heart failure decompensation [11]. EVs are both passive and active players in cardiovascular disease, reflecting the activation of cells of their origin (e.g. platelets or leukocytes) and interacting with other cells, respectively. Recent proteomics study highlighted tissue EVs as important drivers of aortic valve calcification – an important mechanism contributing to AS progression [12]. Circulating EVs increase the adhesion of platelets and deposition of fibrin on human atherosclerotic plaques that increases the risk of thrombus development [13]. Procoagulant properties of EVs are mediated by proteins exposed on their surface, such as PS, tissue factor (TF) or P-selectin, which either directly or indirectly activate the coagulation cascade [14]. Since EVs are established markers of cell activation and involved in vascular homeostasis, concentrations of different subtypes of EVs may serve as a predictor of upcoming cardiovascular events. The objectives of this study were (i) to determine the effect of TAVI on the concentrations of different EVs subtypes and (ii) to evaluate the predictive value of these EVs for MACCE within 1 year after TAVI.

## Methods

### Study Design

This was a prospective study conducted at 3 academic centers in Poland between November 2018 and June 2020, in collaboration with Amsterdam Vesicle Center, Amsterdam University Medical Centers (UMC), the Netherlands. The study protocol was approved by the Ethics Committee of Medical University of Warsaw (approval number: KB/128/2018, KB/4/A2021).

Study population included patients diagnosed with severe AS and qualified for TAVI based on the Heart Team decision, who provided written informed consent to participate in the study. Severe AS was defined according to the recent Guidelines for the management of valvular heart disease as aortic valve area (AVA) < 1.0 cm^2^ or indexed AVA < 0.6 cm^2^/m^2^ as calculated by the continuity equation on transthoracic echocardiography (TTE) [15]. Exclusion criteria were transcatheter valve-in-valve implantation, chronic kidney disease (glomerular filtration rate < 30 mL/min), autoimmune diseases, active neoplastic disease, pregnancy and breast-feeding.

Transcatheter aortic valve implantation was performed by an interventional cardiologist (B.R., J.K., Z.H.) and a cardiac surgeon (R.W.) in a hybrid operating room. Pharmacotherapy after TAVI included single antiplatelet therapy (acetylsalicylic acid or clopidogrel) in patients with no indication for oral anticoagulation (OAC), or OAC if required [16, 17]. Other drugs were continued at the discretion of the treating physician.

Clinical data were collected during the index hospitalization and follow-up visit at 12 ± 3 months after TAVI, when control TTE was performed and data regarding MACCE (all-cause death, cardiovascular death, myocardial infarction, stroke, transient ischemic attack (TIA), decompensation of heart failure or clinical valve thrombosis) were recorded.

The primary endpoint was the predictive value of different EVs subtypes for the occurrence of MACCE during the follow-up period. The secondary endpoint was the difference in plasma expression of EVs before and after TAVI.

### Sample Collection and Handling

Sample collection and handling were done in 3 Polish academic centers by trained professionals (K.P., M.C., O.D., S.J.) according to the to the recent guidelines to study EVs [18]. EV measurements in all samples were done in one block in Vesicle Observation Centre, Amsterdam UMC, following the shipment of all samples on dry ice.

Blood was collected from fasting patients twice into 7.5 mL 0.109 mol/L ethylenediaminetetraacetic acid (EDTA) plastic tubes (S-Monovette, Sarstedt) via antecubital vein puncture: 1 day before TAVI and 5–7 days after TAVI (at hospital discharge). Following preparation of platelet-depleted plasma using double centrifugation (2500 g, 15 min, 20 °C, acceleration speed 1, no brake), samples stored at − 80 °C until analyzed, according to the current guidelines to store biological samples before EV measurements [18]. Prior to analysis, samples were thawed for 1 min in a water bath (37 °C) to avoid cryoprecipitation.

Flow cytometry (A60-Micro, Apogee Flow Systems) was used to determine the concentration of following EV subtypes in platelet-depleted plasma EVs derived from all platelets (CD61 +), activated platelets (CD61 + /CD62p +), leucocytes (CD45 +), erythrocytes (CD235a +) and exposing phosphatidylserine (PS +). To improve the reproducibility of our EV flow cytometry experiments, we (i) applied the framework for standardized reporting of EV flow cytometry experiments (MIFlowCyt-EV) [22] (ii) calibrated all detectors, (iii) determined the EV diameter and refractive index by the flow cytometry scatter ratio (Flow-SR) [20], 20, and (iv) applied custom-built software to fully automate data calibration and processing. All relevant details regarding sample collection and handling, assay controls, instrument calibration, data acquisition, and EV characterization are included in the Supplementary File.

### Statistical Analysis

As there is no data regarding the differences in EV concentrations in patients with and without MACCE, the power calculation for the primary end-point was based on the differences in EV concentrations in patients with calcified AS and healthy controls [23]. Patients with calcified AS had on average twofold higher EV concentrations compared to controls. The required sample size was calculated by a two-sided t-test at a significance level of 0.05 with the following assumptions: (i) mean difference between the groups with and without MACCE = 1.0, (ii) standard deviation (SD) ± 1.0, and (iii) nominal test power = 0.8. Hence, at least 17 patients with MACCE should be enrolled in the study to observe a difference in EV concentrations in patients with or without MACCE. Given that 15% rate of MACCE within a year after TAVI, at least 122 patients should be enrolled.

Statistical analyses were conducted using IBM SPSS Statistics, version 27.0 (IBM, New York, USA). Categorical variables were presented as number and percent and compared using χ2 test. The Shapiro–Wilk test was used to assess normal distribution of continuous variables. Continuous variables were presented as mean with standard deviation (SD) or median with interquartile range (IQR). Changes of EVs concentration before and after TAVI procedure were calculated with Wilcoxon signed-rank test or paired t-test depending on data distribution. To assess the difference in variables between patients with and without MACCE, unpaired t-test or U-Mann Whitney test were used to compare data with and without normal distribution, respectively. Chi-square test was used to compare categorical variables. The predictive value of EVs for MACCE and the cut-offs were calculated using a receiver operating characteristic (ROC) curve. Logistic regression model incorporating EVs concentration and clinical characteristics were used to determine the clinically and statistically optimal model for MACCE. A two-sided p-value below 0.05 was considered significant.

## Results

**Fig. 1:**
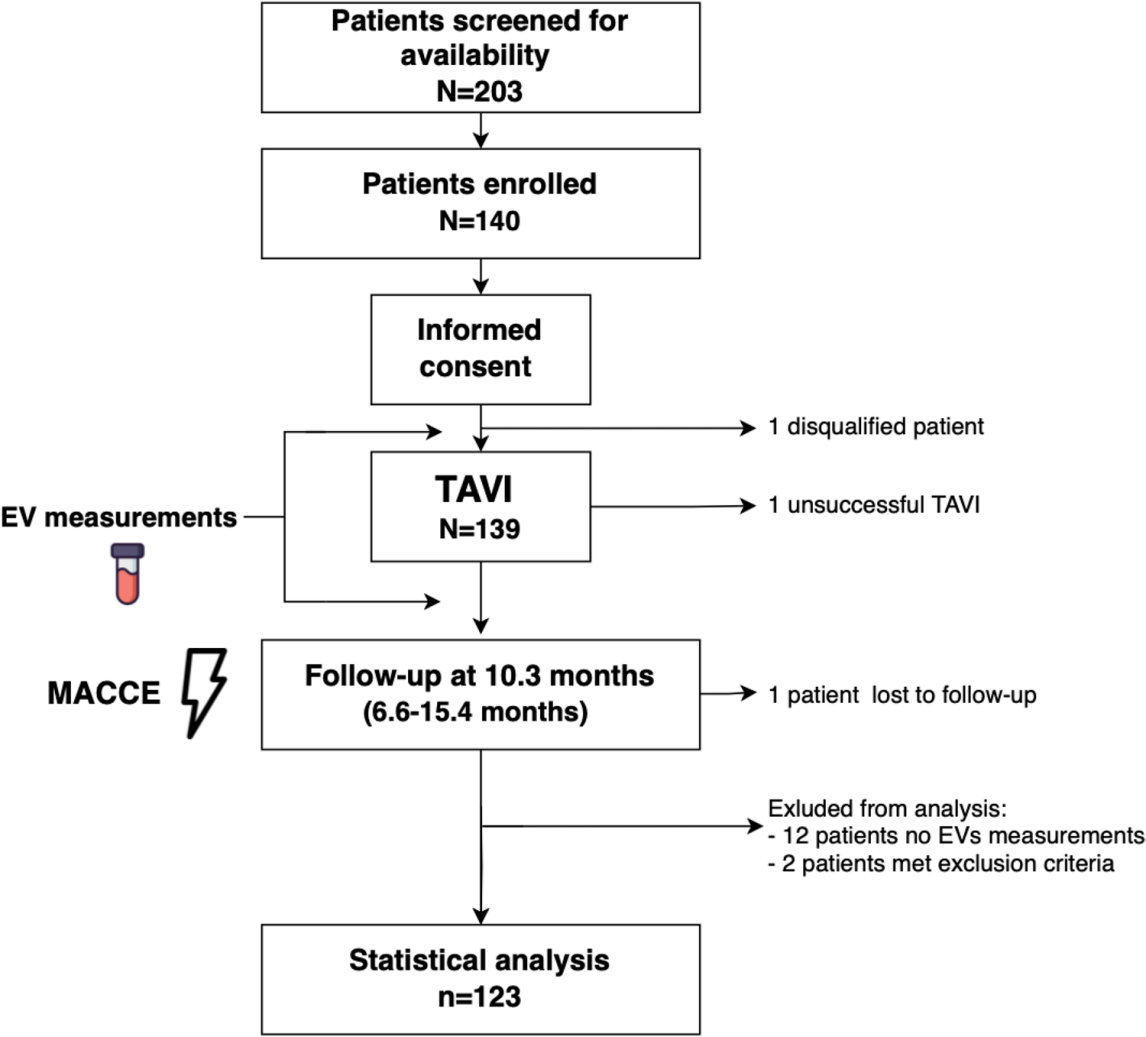
Study design and flow chart; MACCE — major adverse cardiac and cerebrovascular events; TAVI — transcatheter aortic valve implantation

Figure 1 shows the study design and flow chart. Patient characteristics are presented in Table 1. Among 123 patients included in the analysis, 24 (19.5%) patients experienced MACCE during the median follow-up time of 10.3 months (6.6–15.4 months): 5 (20.8%) all-cause deaths, 5 (20.8%) cardiovascular deaths, 1 (4.2%) stroke, 1 (4.2%) TIA, 11 (45.8%) readmissions due to decompensated heart failure and 1 (4.2%) clinical valve thrombosis. Patients who experienced MACCE were older (median age 83.0 vs. 79.0 years, p = 0.006) and more often suffered from COPD (25% vs 8%, p = 0.03). There were no other differences between the groups. The incidence of procedural complications was similar in patients with and without MACCE. At follow-up, the mean LVEF and mean aortic valve gradient were comparable in both groups (60% vs. 55%, p = 0.91 and 8.0 mmHg vs. 9.0 mmHg, p = 0.33, respectively).

**Table 1:** Comparison of baseline characteristics between patients who experienced MACCE and those who did not during a median follow-up of 10.3 months. Number of patients: 123. Statistical tests used: unpaired t-test or U-Mann Whitney test to compare data with and without normal distribution, respectively; Chi-square test to compare categorical variables

	Total population (N = 123)	No MACCE (N = 99)	MACCE (N = 24)	p-value
Baseline characteristics				
Age [years]	80 (74.00–83.00)	79 (74.00–82.00)	83 (79.25–84.00)	**0.006**
Gender. male	56 (45.5%)	44 (44.40%)	12 (50.0%)	0.624
BMI [kg/m2]	26.35 (24.50–29.83)	26.61 (24.50–29.97)	25.83 (23.11–29.67)	0.359
Co-morbidities				
Hypertension	102 (82.90%)	81(79.41%)	21(20.59%)	0.763
Diabetes mellitus	46 (37.70%)	40(40.82%)	6 (25.00%)	0.152
Atrial fibrillation	42(34.10%)	34 (34.34%)	8 (33.33%)	0.925
Prior stroke/TIA	12 (9.80%)	10 (10.10%)	2 (8.33%)	1.000
Prior myocardial infarction	33 (26.80%)	27 (27.27%)	6 (25.00%)	0.822
Prior PCI	54 (43.90%)	43 (43.43%)	11(45.83%)	0.832
Prior CABG	10 (8.10%)	9 (9.09%)	1 (4.17%)	0.685
COPD	14 (11.40%)	8 (8.08%)	6 (25.00%)	**0.030**
Heart failure (NYHA III/IV)	56 (45.52%)	48 (48.48%)	8 (33.33%)	0.181
EuroSCORE II [%]	3.70 (2.45–5.07)	3.71 (2.45–4.92)	3.31 (2.51–5.09)	0.785
CKD > 3a	25 (20.30%)	19 (19.19%)	6 (25.00%)	0.574
Laboratory data				
Hemoglobin [g/d]	11.900 (10.10–13.30)	11.90(9.30–13.30)	11.95 (11.05–13.33)	0.446
Leukocytes (thousand/dl)	6.96 (5.85–8.56)	6.96 (5.84–8.56)	6.97 (6.47–8.55)	0.592
Platelets count (per microliter)	177.00 (153.00–220.00)	179.00 (153.00–223.00)	168.00 (152.25–214.25)	0.431
Creatinine [mg/dL]	1.28 (0.98–1.70)	1.28 (1.00–1.95)	1.26 (0.94–1.44)	0.139
Estimated GFR [mL/min/1.73 m2]	55.00 (43.00–70.00)	56.00 (42.00–70.00)	50.00 (43.00–61.75)	0.350
NT-proBNP (pg/ml)	1863.50 (571.75–3715.25)	2107.50 (564.25–3982.50)	1729.00 (635.50–3307.25)	0.787
CRP	1.20 (0.22–4.65)	1.20 (0.22–4.25)	1.23 (0.24–14.28)	0.557
Echocardiography before TAVI				
Ejection fraction [%]	56.00(46.00–61.50)	55.00 (46.50–60.00)	60.50 (45.25–65.00)	0.101
V max [m/s]	4.2000 (3.80–4.50)	4.20 (3.80–4.50)	4.20 (3.73–4.55)	0.776
Gradient max [mmHg]	71.00 (61.90–82.00)	71.00 (64.00–82.50)	72.00 (45.75–81.75)	0.369
Gradient mean [mmHg]	42.22 (33.25–51.00)	43.00 (34.70–52.50)	41.00 (27.00–45.00)	0.077
AVA (VTI) [cm^2^]	0.78 (0.64–0.87)	0.78 (0.62–0.86)	0.80 (0.64–0.90)	0.836
AVAi [cm^2^/m^2^]	0.42 (0.35–0.48)	0.42 (0.35–0.48)	0.48 (0.35–0.52)	0.238
Low-flow. low-gradient AS	28 (22.76%)	19 (20.65%)	9 (39.13%)	0.065
Procedural characteristics				
Access site:				0.102
Femoral	119 (96.75%)	97 (97.98%)	22 (91.67%)	
Subclavian	1 (0.81%)	1 (1.01%)	0 (0.00%)	
Carotid	3 (2.44%)	1 (1.01%)	2 (8.33%)	
Prosthesis size [mm]:				0.402
20	1 (0.81%)	1 (1.01%)	0 (0.00%)	
22	2 (1.62%)	2 (2.02%)	0 (0.00%)	
23	7 (5.7%)	3 (2.03%)	4 (16.67%)	
24	4 (3.25%)	4 (4.04%)	0 (0.00%)	
25	27 (21.95%)	21 (21.21%)	6 (25.00%)	
26	9 (7.32%)	8 (8.08%)	1 (4.17%)	
27	16 (13.00%)	11 (11.11%)	5 (20.83%)	
29	28 (22.76%)	24 (24.24%)	4 (16.67%)	
34	26 (21.14%)	22 (22.22%)	4 (16.67%)	
Missing data	3 (2.44%)	3 (3.03%)	0 (0.00%)	
Valve type:				0.685
EvolutR	40 (32.52%)	35 (35.35%)	5 (20.83%)	
EvolutPRO	12 (9.76%)	9 (9.09%)	3 (12.50%)	
Portico	35 (28.46%)	24 (24.24%)	11 (45.83%)	
Accurate_Neo	10 (8.13%)	8 (8.08%)	2 (8.33%)	
Accurate_Neo2	24 (19.51%)	21 (21.21%)	3 (12.50%)	
Hydra	1 (0.81%)	1 (1.01%)	0 (0.00%)	
Navitor	1 (0.81%)	1 (1.01%)	0 (0.00%)	
Procedure complications				
Life-threatening or disabling bleeding*	11 (8.94%)	8 (8.08%)	3 (12.50%)	0.447
Surgical intervention at access site	8 (6.5%)	6 (6.06%)	2 (8.33%)	0.653
Echocardiography at follow-up				
Ejection fraction [%]	60.00(49.50–60.00)	60.00 (50.00–60.00)	55.00 (42.50–64.00)	0.913
V max [m/s]	2.01 (+ -0.44)	2.00 (+ -0.45)	2.05 (+ -0.40)	0.685
Peak AV gradient [mmHg]	17.43 (+ -6.98)	17.30 (+ -7.12)	18.21 (+ -6.26)	0.645
Mean AV gradient [mmHg]	8.00 (6.00–10.00)	8.00 (6.00–10.25)	9.00 (7.00–10.50)	0.334
AVA (VTI) [cm^2^]	1.92 (+ -0.41)	1.93 (+ -0.42)	1.881875 (+ -0.37)	0.690
AVAi [cm^2^/m^2^]	1.02 (0.94–1.18)	1.01 (0.93–1.18)	1.12 (0.93–1.20)	0.355
Paravalvular leak ≥ moderate	9 (7.32%)	8 (8.08%)	1 (4.17%)	0.509
Post-TAVI procedure concomitant medications				
Beta-blockers	97 (78.86%)	81 (84.38%)	16(76.19%)	0.353
ACE inhibitors	74 (60.16%)	59 (60.20%)	15(71.43%)	0.336
MRA	32 (26.01%)	29 (30.21%)	3(15.00%)	0.166
Diuretics	101 (82.11%)	81 (82.65%)	20 (95.24%)	0.192
Statins	102 (82.93%)	83 (85.57%)	19 (90.48%)	0.734
Proton pump inhibitors	92 (74.80%)	74 (76.29%)	18 (85.71%)	0.561
Acetylsalicylic acid	81 (65.85%)	70 (71.43%)	11(52.38%)	0.089
P2Y12 inhibitor	88 (71.54%)	74 (76.29%)	14 (66.66%)	0.359
Anticoagulant	52 (42.27%)	42 (42.42%)	10 (41.67%)	0.946

^*^According to the Valve Academic Research Consortium (VARC). Bold p value indicates significantly different (< 0.05). Data are shown as

number (percentage). median (interquartile range). mean ± standard deviation; ACE — angiotensin-converting enzyme;

AVA — aortic valve area; AVAi — aortic valve area index; CABG — coronary artery bypass graft surgery; COPD —

chronic obstructive pulmonary disease; CKD — chronic kidney disease; GFR — glomerular filtration rate; CRP- C-reactive protein. MACCE — major adverse cardiac and cerebrovascular events; MRA — mineralocorticoid receptor antagonists; NT-proBNP — N-terminal pro B natriuretic peptide; NYHA — New York Heart Association; PCI — percutaneous coronary intervention; TIA — transient ischemic attack

**Fig. 2:**
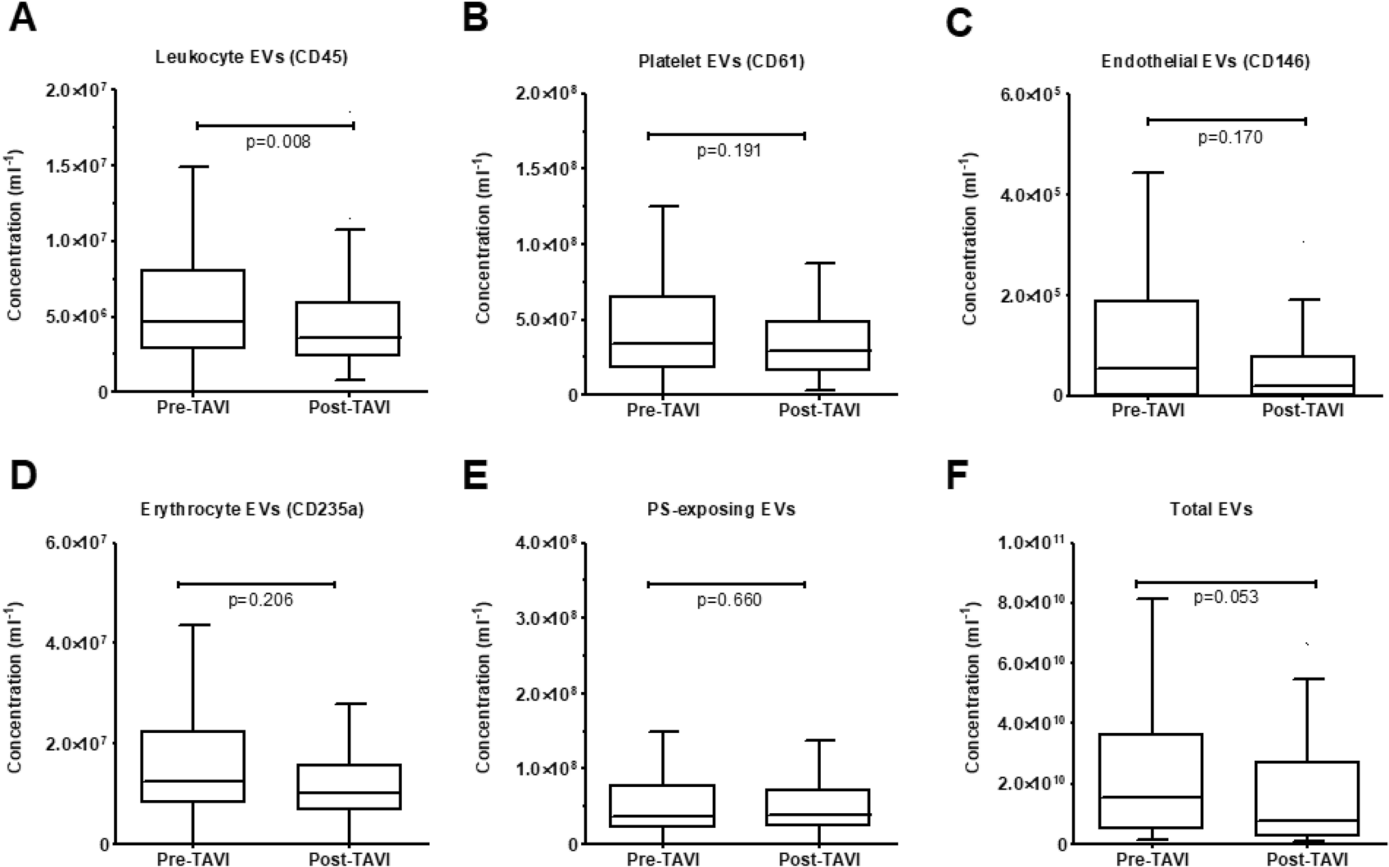
Comparison of plasma extracellular vesicles (EVs) concentrations before and after transcatheter aortic valve implantation (TAVI). Concentration of EVs from leucocytes were lower after TAVI, compared to the measurement before (panel A). There was a trend towards lower concentrations of total EVs after TAVI (panel F). There were no significant differences in the concentrations of other EV subtypes. Number of patients: 123. Statistical tests used: paired t-test or Wilcoxon signed-rank test to compare data with and without normal distribution, respectively

Concentration of EVs from leukocytes (CD45 +) were lower after TAVI compared to bassline (p = 0.008; Fig. 2). There was a trend towards lower concentrations of total EVs after TAVI (p = 0.053; Fig. 2). We found no significant differences in the concentrations of other EV subtypes (p ≥ 0.170 for all) (Fig. 2, Supplementary Table 1). When analyzing the changes in percentage of different EVs subtypes compared to total EV concentrations before and after TAVI, we found no significant differences (Supplementary Fig. 1).

**Fig. 3:**
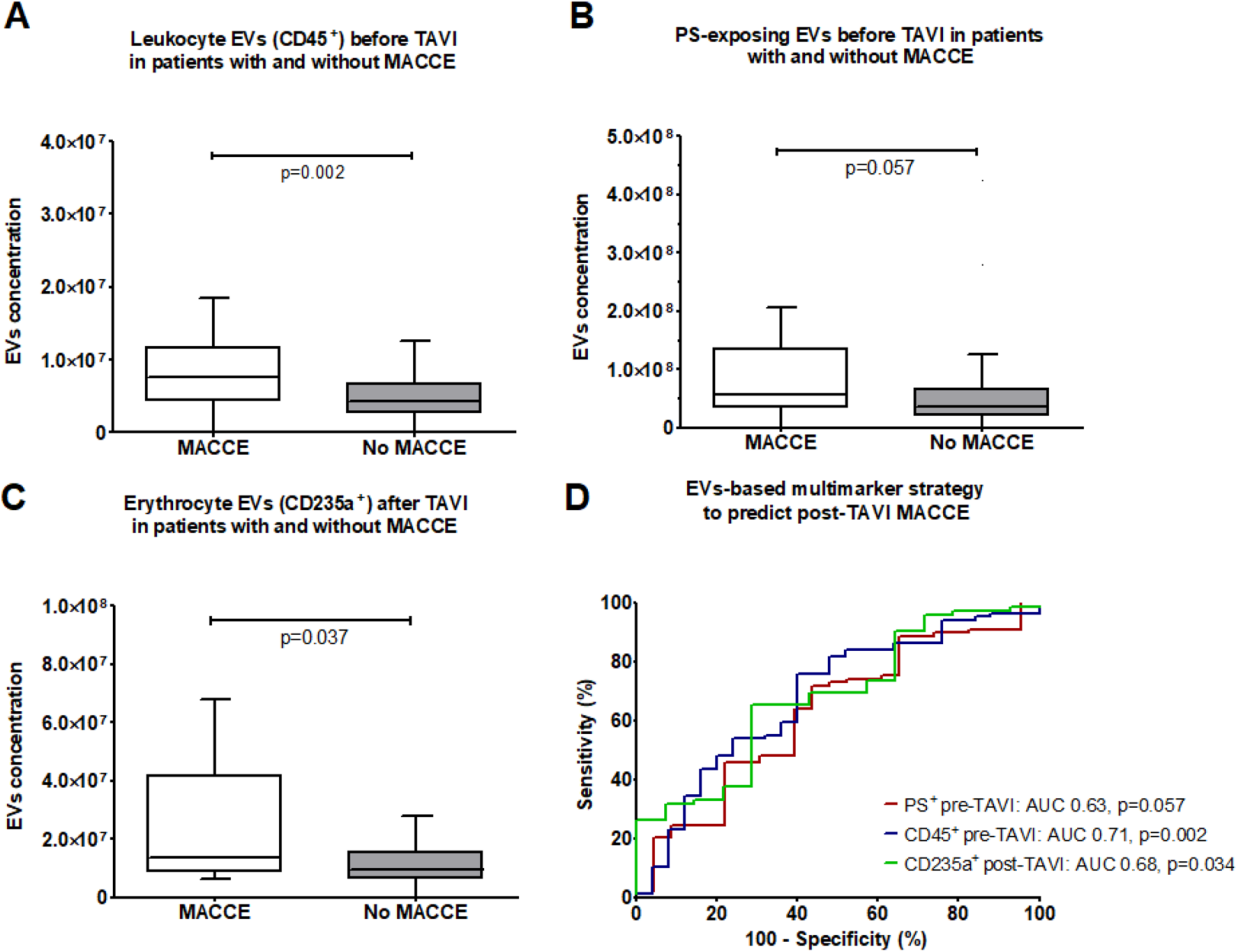
Pre-TAVI concentrations of EVs from leukocytes (A) and EVs exposing PS (B), and post-TAVI concentrations of EVs from erythrocytes (C) in patients who did and did not experience MACCE during follow-up period with ROC curves for prediction of MACCE (D). Number of patients: 123. Statistical tests used: U-Mann Whitney test, receiver operating characteristic (ROC) analysis

Baseline concentrations of leukocyte EVs (CD45 +) was higher in patients who experienced MACCE, compared to those who did not (p = 0.002; Fig. 3A) and discriminated between these two groups of patients (area under ROC curve (AUC) = 0.707, p = 0.002; Fig. 3B). There was a trend toward higher concentrations of pre-TAVI PS-exposing EVs (PS +), and post-TAVI EVs from erythrocytes (CD235a +) in patients who experienced MACCE (p = 0.057, p = 0.034, respectively; Fig. 3C-F). Concentrations of other analyzed subtypes of EVs did not differ among patients with and without MACCE (Supplementary Table 2).

Table 2 shows the statistical estimates for the prediction of MACCE by pre-TAVI concentrations of EVs from leukocytes (CD45 +) and EVs exposing PS and post-TAVI concentrations of EVs from erythrocytes (CD235a +), determined based on the ROC curve. In univariable analysis, MACCE were predicted by pre-TAVI concentrations of leukocyte EVs (62.5% sensitivity, 76.1% specificity), pre-TAVI concentrations of PS + EVs (78.3% sensitivity, 46.1% specificity) and post-TAVI concentrations of erythrocyte EVs (71.4% sensitivity and 65.2% specificity). To check whether these three EVs were independent predictors of MACCE, they were incorporated in multivariable Cox regression analyses (Table 3, Supplementary Table 3). Patients with increased pre-TAVI concentration of PS-exposing EVs had over fivefold higher odds of MACCE after TAVI, independent of other clinical variables (odds ratio [OR] 5.313, 95% confidence interval [CI] 1.164–24.258, p = 0.031). High baseline pre-TAVI concentrations of leukocyte EVs or post-TAVI concentrations of erythrocytes EVs did not predict MACCE in multivariable analyses (p = 0.532 and p = 0.391, respectively).

**Table 2:** Statistical estimates for prediction of MACCE by subtypes of EVs which significantly differed among patients with and without MACCE. Number of patients: 123. Statistical test used: receiver operating characteristic (ROC) analysis

EVs	AUC (95% CI)	p-value	Cut-off (particles * × 10^6^ per mL plasma)	Sensitivity	Specificity	PPV	NPV	PLR
Pre-TAVI CD45 +	0.71 (0.59–0.82)	0.002	6.73	62.5%	76.1%	41.7%	88.2%	2.62
Pre-TAVI PS +	0.63 (0.50–0.76)	0.057	32.5	78.3%	46.1%	27.3%	89.1%	1.45
Post-TAVI CD235a +	0.68 (0.53- 0.83)	0.034	12.7	71.4%	65.3%	28.6%	92.2%	2.06

AUC — area under the curve; CI — confidence interval; EVs – extracellular vesicles; MACCE – major adverse cardiac and cerebrovascular events; PPV — positive predictive value; NPV — negative

predictive value; PLR — positive likelihood ratio

**Table 3:** Multivariable Cox regression analysis for prediction of MACCE by subtypes of EVs which significantly differed among patients with and without MACCE, after adjustment for sex, age, chronic obstructive pulmonary disease, mean gradient, low-flow and low-gradient aortic stenosis and acetylsalicylic acid use. Number of patients: 123

EVs	Cox regression	OR	95% CI		P
			Lower	Upper	
High pre-TAVI CD45 + EVs concentration (cut-off > 6.73*10^6^ mL^−1^)	Univariable	2.545	1.080	5.995	0.033
	Multivariable*	1.409	0.480	4.134	0.532
High pre-TAVI PS + EVs concentration (cut-off > 32.54*10^6^ mL^−1^)	**Univariable**	**2.855**	**0.964**	**8.456**	**0.058**
	Multivariable*	**5.313**	**1.164**	**24.258**	**0.031**
High post-TAVI CD235a + EVs concentration (cut-off > 12.75 *10^6^ mL^−1^)	Univariable	2.914	0.877	9.682	0.081
	Multivariable*	1.783	0.476	6.671	0.391

EVs – extracellular vesicles; MACCE- major adverse cardiac and cerebrovascular events

Bold p value indicates significantly different (< 0.05)

**Fig. 4:**
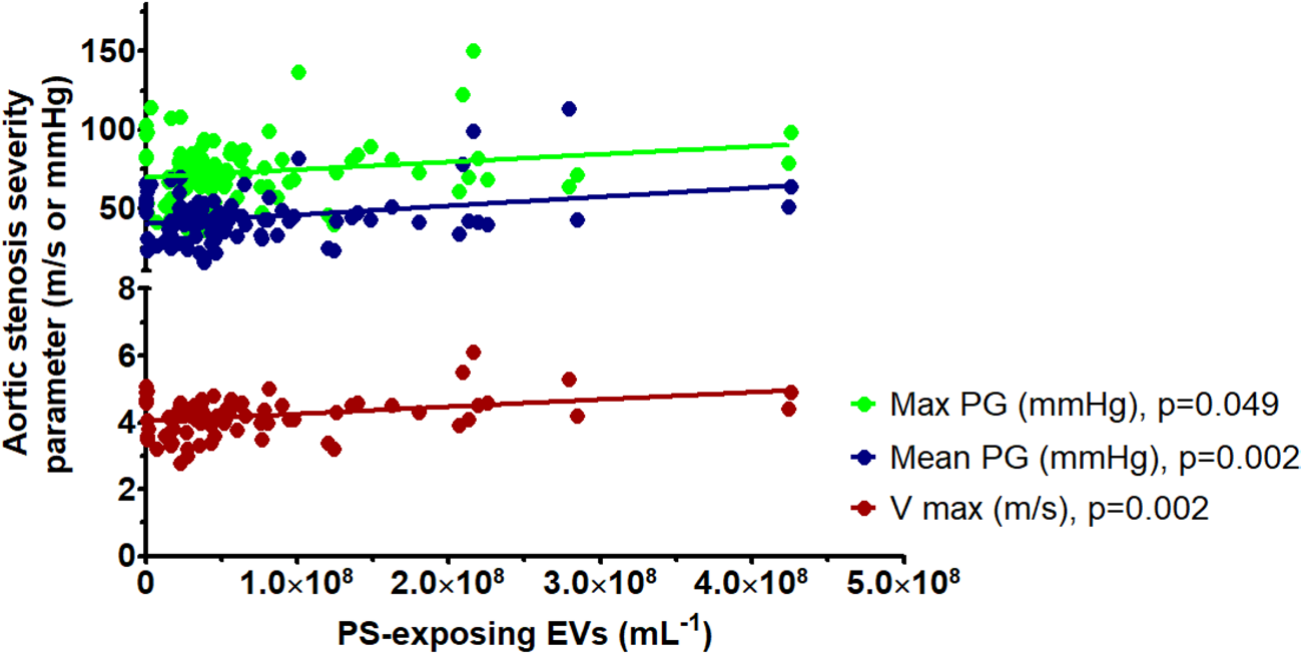
Correlation between plasma PS-exposing EV concentrations and aortic blood flow parameters, assessed in echocardiography before TAVI. Max PG – peak aortic valve gradient, mean PG – mean aortic valve gradient, V max – peak aortic valve velocity. Number of patients: 123. Statistical test used: Spearman's rank correlation coefficient

There was a positive correlation between baseline PS-exposing EV concentrations and (i) peak aortic valve velocity, (ii) peak aortic valve gradient and (iii) mean aortic valve gradient (p ≤ 0.049 for all) (Fig. 4). There were no significant correlations between PS-exposing EV concentrations and AVA, AVAi, EF and NT-proBNP (≥ 0.055 for all, Supplementary Table 4).

**Fig. 5:**
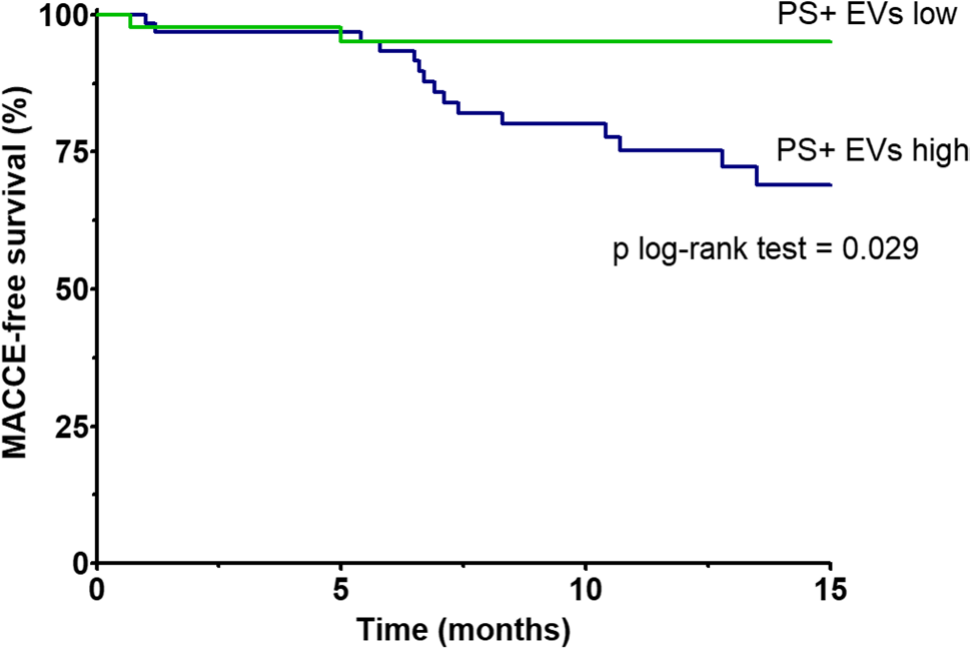
Kaplan–Meier survival analysis for MACCE after TAVI patients with high or low concentration of PS-exposing EVs, which was the only predictor of MACCE in multivariable analysis. Number of patients: 123. Statistical test used: log-rank test

**Fig. 6:**
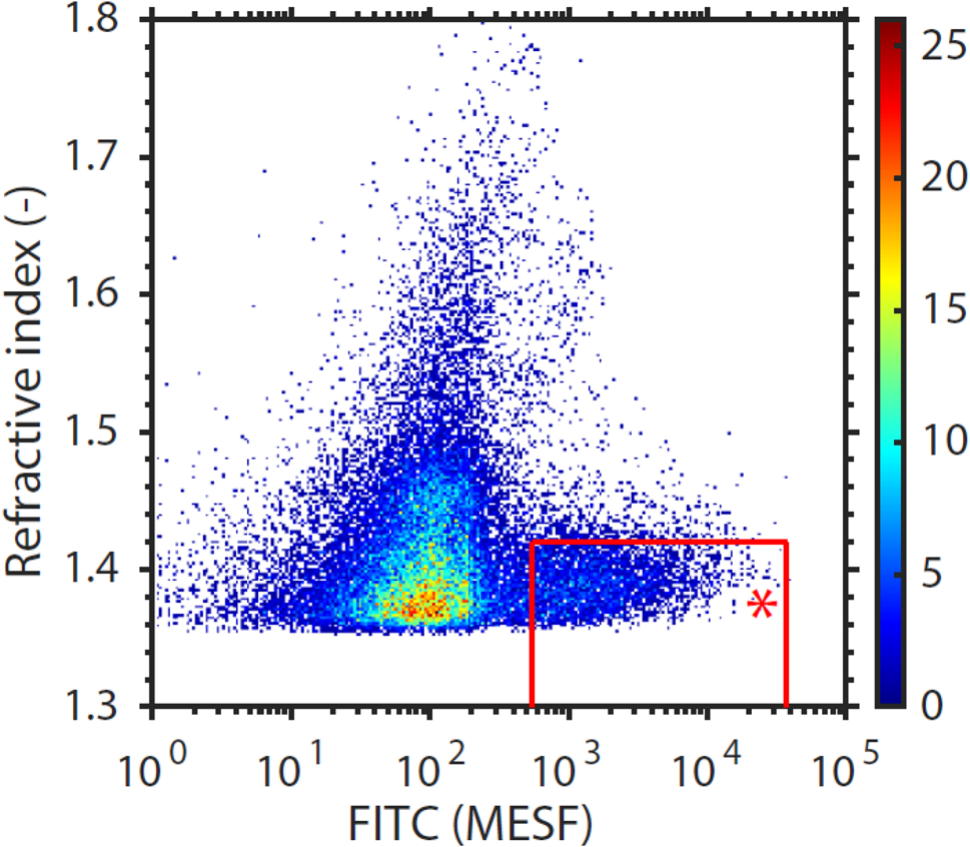
A representative flow cytometry chart (A60-Micro, Apogee Flow Systems) showing phosphatidylserine-exposing EVs positive at the fluorescence detector corresponding to fluorescein isothiocyanate (FITC) EVs in platelet-depleted plasma (red gate with red star). The FITC fluorescence is expressed in standard units of the number of molecules of equivalent soluble fluorochrome (MESF). Particles left to the red gate are negative for FITC and correspond to the background noise level of the FITC detector. Particles above the red gate have a refractive index > 1.42, likely corresponding to positively labeled chylomicrons. The gated events are related to plasma concentration by taking into account the sample dilution, flow rate and measurement time

Kaplan–Meier analysis of event-free survival for MACCE in patients after TAVI (Fig. 5) demonstrated that patients with high concentration of PS-exposing EV concentrations (defined as > 32.54 *10^6^ per mL plasma based on the ROC curve) had a lower chance of event-free survival during follow-up, compared to patients with concentrations of PS-exposing EV below the cut-off concentration (p = 0.048 for the log-rank test). A representative flow cytometry chart showing PS-exposing EVs in plasma is shown in Fig. 6.

## Discussion

To our best knowledge, this is the first prospective, multicenter study investigating the effect of TAVI on plasma EV concentrations and providing a prediction model for post-TAVI MACCE based on EV concentration analysis. In addition, this is the first multicenter study, where the framework for standardized reporting of EV flow cytometry experiments (MIFlowCyt-EV) has been applied to improve the reproducibility of EV flow cytometry experiments. The main findings of our study are that (i) TAVI leads to a decrease in plasma concentration of EVs originating from leukocytes (CD45 +), and (ii) patients with increased pre-TAVI concentration of PS-exposing EVs have over fivefold higher odds of adverse post-TAVI outcomes, independent of other clinical variables, during the median observation time of over 10 months.

Obstruction of blood outflow leads to pressure overload-induced heart failure with subsequent release of EVs from activated blood cells and endothelium [23–25]. TAVI restores normal hemodynamic conditions, as reflected by decreased activation and suppressed pro-inflammatory and pro-atherogenic properties of monocytes [26]. In our study, we also showed that TAVI decreases leukocytes activation, reflected by decreased EVs release, which supports the previously observed anti-inflammatory effect of TAVI.

Reports regarding the effect of TAVI on the concentrations of other blood cells- and endothelial cell-derived EVs are inconsistent. In two studies including 92 and 9 patients with severe AS, TAVI had no effect on platelet- and endothelial-derived EVs 5–7 days after the procedure [27, 28]. Another study showed an increase in platelet-derived and PS-exposing EV concentrations, along with a decrease in endothelial-derived EV concentrations 7 days after TAVI [29]. Two studies showed a decrease in endothelial EVs concentrations 3–6 months after TAVI [29, 30], which may indicate restoration of endothelial integrity following the correction of vascular hemodynamics in a long-term observation [31]. These inconsistent results might be explained by different timepoints of EV measurements after TAVI, and different antibody subtypes to detect endothelial and platelet-derived EVs, hampering head-to-head comparisons. It could be also speculated that the rapid decrease in leukocyte EV concentration after TAVI is due to the fact that leukocytes (specifically monocytes) (i) are the largest blood cells and thereby most susceptible to shear stress caused by aortic stenosis and (ii) have exceptional adhesive properties that interact with stenotic valve, leading to leukocytes activation and release of EVs. Finally, the post-TAVI decreased in leukocyte EV concentrations might be not due to decreased EV release, but due to increased EV clearance. For example, if more PS is exposed on EVs, which is recognized as an “eat-me signal” [32], the leukocyte EV clearance would be faster. Although we have not seen differences in PS-exposing EV concentrations after TAVI, other authors observed a gradual increase in PS-exposing EVs up to 6 months post-TAVI, which might explain the decrease in the concentration of other EV subtypes [29].

We found that increased pre-TAVI concentration of PS-exposing EVs predict post-TAVI MACCE. PS exposed on the membrane surface binds clotting factors and further propagate thrombin generation [33]^33^. Hence, higher concentrations of PS-exposing EVs reflect increased ability to promote coagulation, which in our study was reflected by more MACCE. A recent study also showed that the concentration of PS-exposing EVs increases gradually in the first 6 months after TAVI, which was associated with higher serum coagulation activity in vivo [29], supporting our results that the excessive release of circulating PS-exposing EVs contributes to higher incidence of post-TAVI MACCE. We did not observe a higher prevalence of thrombosis-related MACCE, including MI, TIA, stroke or valve thrombosis among patients with high pre-TAVI PS-exposing EVs concentration. Nevertheless, given relatively small number of thrombosis-related MACCE in our study, such association cannot be excluded and should be investigated in a larger group of patients. We showed that the concentrations of PS-exposing EVs are higher in patients with more severe aortic blood flow disturbances, based on echocardiographic parameters – mean and peak aortic gradient pressure and peak aortic gradient velocity. Interestingly, the concentration of these EVs did not correlate with morphological severity of AS, as assessed by AVA and AVAi. It could be speculated that excessive release of PS-exposing EVs results from shear stress, which might be reflecting AS progression more accurately than echocardiographic examination.

Identification of patients at high risk of adverse outcomes after TAVI becomes an increasing clinical challenge, especially given the expansion of TAVI to young patients at intermediate and low perioperative risk [35, 36]. It is important for optimization of antithrombotic and/or anticoagulation strategies. Future possible targeted therapies, such as inhibition of PS-dependent hypercoagulable state might become a milestone of long-term care of patients after TAVI. The associated between higher baseline concentration of procoagulant PS-exposing EVs and MACCE might bring a rationale for administration of antiplatelet and/or anticoagulant therapy before TAVI procedure in a subset of patients. Currently, monotherapy with a single antiplatelet drug (aspirin or clopidogrel) is the standard care in post-TAVI patients without an indication for oral anticoagulation. However, aspirin does not decrease the concentrations of plasma EVs, whereas clopidogrel seems to have only minor effect on EVs [37]. In contrast, a potent P2Y12 receptor inhibitor ticagrelor was shown to decrease the concentrations of PS-exposing EVs more than clopidogrel [37]. From the pathophysiological point of view, ticagrelor might be a viable option after TAVI, especially in the subgroup of patients with high baseline concentrations of PS-exposing EVs. This interesting hypothesis remains to be investigated in future clinical studies to establish evidence-based recommendations.

## Limitations

There are limitations of this study, which should be acknowledged. First of all, the number of thrombosis-associated MACCE is our study was too low to reach statistical power in subanalysis regarding the association between PS-exposing EV and individual MACCE events. Secondly, the aim of study was to investigate the effect of TAVI on EVs concentration and an association between EV concentration and MACCE, so no platelet function tests or coagulation activity tests were performed to confirm the functionality of PS-exposing EVs in vivo, demonstrated in other studies [29]. Third, all TAVI procedures were done by the same team, which eliminated the bias due to various expertise levels, but also limited the general results applicability. Fourth, many previous studies showed that PS-exposing EVs are elevated in cardiovascular diseases, limiting the specificity of our finding. However, considering the fact that the biomarkers which are currently most widely established in cardiovascular disease such as D-dimer and cardiac troponin also have low specificity, this limitation does not exclude the diagnostic utility of PS-exposing EVs in patients undergoing TAVI. Finally, PS exposure might be an artifact related to presence of platelets and/or cells fragmented during centrifugation and freeze-thawing. Although we did not study our plasma samples with electron microscopy, the standardized pre-analytical and analytical protocols, partly developed by our research group and applied in this study [18–20, 22, 38], were used to maximize the quality and reliability of the results.

## Conclusions

Patients with increased pre-TAVI concentration of procoagulant, PS-exposing EVs have over fivefold higher odds of adverse outcomes after TAVI. The next step is to conduct a multicenter trial specifically focusing on PS-exposing EVs to predict post-TAVI MACCE.

## Supplementary Information

Below is the link to the electronic supplementary material.

Supplementary file1 (DOCX 73.2 KB)

Supplementary file2 (DOCX 357 KB)

## Data Availability

Source data are available upon request to the corresponding author.

## Abbreviations

ACE: Angiotensin-converting enzyme
AS: Aortic stenosis
AVA: Aortic valve area
AVAi: Aortic valve area index
AUC: Area under the curve
CI: Confidence interval
CABG: Coronary artery bypass graft surgery
COPD: Chronic obstructive pulmonary disease
CKD: Chronic kidney disease
CRP: C-reactive protein
GFR: Glomerular filtration rate
EVs: Extracellular vesicles
LVEF: Left ventricle ejection fraction
MACCE: Major adverse cardiac and cerebrovascular events
MRA: Mineralocorticoid receptor antagonists
NPV: Negative predictive value
NT-proBNP: N-terminal pro B natriuretic peptide
NYHA: New York Heart Association
PCI: Percutaneous coronary intervention
PLR: Positive likelihood ratio
PG: Pressure gradient
SVI: Stroke volume index
PS: Phosphatidylserine
PPV: Positive predictive value
STS: Society of Thoracic Surgeons
SAVR: Surgical aortic valve replacement
SD: Standard deviation
TF: Tissue factor
TIA: Transient ischemic attack
TAVI: Transcatheter aortic valve implantation
TTE: Transthoracic echocardiography
VARC: Valve Academic Research Consortium

## Declarations

All procedures followed were in accordance with the ethical standards of the responsible committee on human experimentation (institutional and national) and with the Helsinki Declaration of 1975, as revised in 2000. Informed consent was obtained from all patients for being included in the study.
