# Supplementary material for: Extracellular Vesicles to Predict Outcomes After Transcatheter Aortic Valve Implantation – a Prospective, Multicenter Cohort Study
Source: J Cardiovasc Transl Res. 2024 May 28;17(5):992–1003. doi: 10.1007/s12265-024-10521-x (PMC11519094; doi:10.1007/s12265-024-10521-x)
Supplement: Supplementary file 1 — Supplementary file1 (DOCX 73.2 KB) [file 12265_2024_10521_MOESM1_ESM.docx]

**Extracellular vesicles to predict outcomes after transcatheter**

**aortic valve implantation – a prospective, multicenter cohort study**

**Supplementary Materials**

*Table S1. Comparison of plasma extracellular vesicles (EVs) concentrations before and after transcatheter aortic valve implantation (TAVI). EVs concentrations presented as number of particles x10^6^ per mL plasma. Number of patients: 123. Statistical tests used: paired t-test or Wilcoxon signed-rank test to compare data with and without normal distribution, respectively.*

| EV type | Pre-TAVI (*10^6^ mL^-1^) | Post-TAVI (*10^6^ mL^-1^) | p-value |
| --- | --- | --- | --- |
| Total | 15596.19 (4692.83-36184.90) | 7662.78 (2667.07-27139.35) | **0.053** |
| CD45+ | 4.71 (2.83-8.07) | 3.64 (2.39-5.89) | **0.008** |
| CD146+ | 0.05 (0.00-0.19) | 0.02 (0.00-0.08) | 0.169 |
| CD61+ | 33.85 (17.35-65.05) | 28.93 (15.87-48.34) | 0.190 |
| CD61+CD62p+ | 0.03 (0.00-0.12) | 0.01 (0.00-0.11) | 0.208 |
| CD235a+ | 12.65 (8.14-22.51) | 10.25 (6.59-15.78) | 0.205 |
| PS+ | 37.83 (21.52-76.77) | 39.14 (22.53 -71.11) | 0.658 |

*Table S2. Concentrations of extracellular vesicles (EVs) in patients who experienced MACCE*

*and those who did not during a median follow-up of 10.3 months. Number of patients: 123. Statistical tests used: U-Mann Whitney test.*

|  | Total population (n=123) | No MACCE (n=99) | MACCE (m=24) | p-value |
| --- | --- | --- | --- | --- |
| **Pre-TAVI (x10^6^ per mL plasma)** | | | | |
| \| Total \| \| --- \| \|  \| | 15596.19 (4692.83-36184.90) | 14660.26 (4392.23-36725.23) | 19157.62 (7641.88-36249.59) | 0.430 |
| CD45+ | 4.71 (2.83-8.07) | 4.29 (2.64-6.66) | 8.03 (4.52-12.48) | **0.002** |
| CD146+ | 0.05 (0.00-0.19) | 0.05 (0.0-0.19) | 0.10 (0.07-0.20) | 0.287 |
| CD61+ | 33.85 (17.35-65.05) | 31.45 (17.35-55.90) | 41.47 (17.03-81.71) | 0.621 |
| CD61+CD62p+ | 0.03 (0.00-0.12) | 0.02 (0.00-0.09) | 0.05 (0.00-0.12) | 0.819 |
| CD235a+ pre-TAVI | 12.65 (8.14-22.51) | 12.31 (8.11-17.98) | 18.14 (8.43-26.21) | 0.125 |
| PS+ pre-TAVI | 37.83 (21.52-76.77) | 36.68 (19.19-67.17) | 56.28 (32.89-136.09) | **0.057** |
| **Post-TAVI (x10^6^ per mL plasma)** | | | | |
| Total | 7662.78 (2667.07-27139.35) | 7162.63 (2677.31-28497.15) | 9703.27 (2541.75-19090.31) | 0.760 |
| CD45+ | 3.64 (2.39-5.89) | 3.61 (2.22-6.15) | 3.81 (2.63-4.94) | 0.885 |
| CD146+ | 0.02 (0.00-0.08) | 0.02 (0.00-0.07) | 0.04 (0.00-0.18) | 0.508 |
| CD61+ | 28.93 (15.87-48.34) | 28.63 (15.65-48.42) | 29.24 (15.91-45.43) | 0.966 |
| CD61+CD62p+ | 0.01 (0.00-0.11) | 0.01 (0.00-0.10) | 0.05 (0.01-0.13) | 0.180 |
| CD235a+ | 10.25 (6.59-15.78) | 9.38 (6.33-15.39) | 13.63 (8.46-41.74) | **0.034** |
| PS+ | 39.14 (22.53 -71.11) | 39.10 (21.73-66.90) | 43.20 (35.55-80.32) | 0.325 |

*Table S3. Multivariable Cox regression analysis for prediction of MACCE subtypes of EVs which significantly differed among patients with and without MACCE. Number of patients: 123.*

| Variable | OR | 95% CI | | p-value |
| --- | --- | --- | --- | --- |
|  |  | Lower  | Upper |  |
| High pre-TAVI EVs from leukocytes (CD45+) | 1.409 | 0.480421 | 4.133776 | 0.532 |
| Age | 1.112 | 0.997 | 1.241 | 0.056 |
| Sex (male) | 0.990 | 0.370 | 2.652 | 0.985 |
| COPD | 2.696 | 0.845 | 8.602 | 0.094 |
| ASA use | 0.583 | 0.226 | 1.502 | 0.264 |
| Mean gradient in ECHO before procedure | 0.953 | 0.903 | 1.007 | 0.085 |
| Low-flow and low-gradient AS | 0.927 | 0.291 | 2.958 | 0.899 |

| Variable | OR | 95% CI | | p-value |
| --- | --- | --- | --- | --- |
|  |  | Lower  | Upper |  |
| High pre-TAVI PS-exposing EVs | 5.313 | 1.164 | 24.258 | 0.031 |
| Age | 1.108 | 0.988 | 1.243 | 0.080 |
| Sex (male) | 1.373 | 0.505 | 3.734 | 0.535 |
| COPD | 4.141 | 1.298 | 13.212 | 0.016 |
| ASA use | 0.733 | 0.261 | 2.061 | 0.556 |
| Mean gradient in ECHO before procedure | 0.952 | 0.905 | 1.003 | 0.062 |
| Low-flow and low-gradient AS | 0.806 | 0.244 | 2.663 | 0.724 |

| Variable | OR | 95% CI | | p-value |
| --- | --- | --- | --- | --- |
|  |  | Lower  | Upper |  |
| High post-TAVI EVs from erythrocytes (CD235a+) | 1.783 | 0.476 | 6.671 | 0.391 |
| Age | 1.071 | 0.930 | 1.234 | 0.341 |
| Sex (male) | 1.220 | 0.375 | 3.967 | 0.741 |
| COPD | 6.300 | 1.230 | 2.273 | 0.027 |
| ASA use | 0.369 | 0.104 | 1.303 | 0.122 |
| Mean gradient in ECHO before procedure | 0.962 | 0.905 | 1.023 | 0.214 |
| Low-flow and low-gradient AS | 1.655 | 0.344 | 7.962 | 0.529 |

*Table S4. Correlation between pre-TAVI PS-exposing EVs concentration and echocardiographic and laboratory parameters measured before TAVI in the study group. Number of patients: 123. Statistical test used: Spearman's rank correlation coefficient.*

| Echocardiographic parameter correlated with pre-TAVI PS+ EVs concentration | Pearson correlation | p-value (two-sided) | 95% CI | |
| --- | --- | --- | --- | --- |
|  |  |  | Lower | Upper |
| Ejection fraction (%) | 0.182 | 0.055 | -0.004 | 0.357 |
| Peak aortic valve velocity (m/s) | 0.335 | **0.002** | 0.130 | 0.513 |
| Peak aortic valve gradient (mmHg) | 0.197 | **0.049** | 0.001 | 0.378 |
| Mean aortic valve gradient | 0.297 | **0.002** | 0.112 | 0.461 |
| Aortic valve area, AVA (cm^2^) | -0.031 | 0.769 | -0.237 | 0.177 |
| Aortic valve area index, AVAi (cm^2^/m^2^) | -0.111 | 0.313 | -0.316 | 0.105 |
| N-terminal pro B-type natriuretic peptide concentration, NT-pro-BNP (pg/ml) | -0.008 | 0.941 | -0.206 | 0.191 |
