# Supplementary material for: Extracellular Vesicles to Predict Outcomes After Transcatheter Aortic Valve Implantation – a Prospective, Multicenter Cohort Study
Source: J Cardiovasc Transl Res. 2024 May 28;17(5):992–1003. doi: 10.1007/s12265-024-10521-x (PMC11519094; doi:10.1007/s12265-024-10521-x)
Supplement: Supplementary file 2 — Supplementary file2 (DOCX 357 KB) [file 12265_2024_10521_MOESM2_ESM.docx]

MIFlowCyt-EV of study ““Extracellular vesicles to predict outcomes after transcatheter aortic valve implantation – a prospective, multicenter cohort study”

This document aims to provide the minimum information required to reproduce the flow cytometry experiments on extracellular vesicles (EVs) performed in the study “Extracellular vesicles to predict outcomes after transcatheter aortic valve implantation – a prospective, multicenter cohort study”. This document is based on three published standardization frameworks and guidelines [1–3].

**Contents**

[1 Experiment overview 3](#_Toc157947370)

[1.1 Contact details 3](#_Toc157947371)

[1.1.1 Experiment leader 3](#_Toc157947372)

[1.1.2 Flow cytometry execution 3](#_Toc157947373)

[1.2 Purpose 3](#_Toc157947374)

[1.3 Keywords 3](#_Toc157947375)

[1.4 Experiment variables 3](#_Toc157947376)

[1.5 Experiment design and quality controls 3](#_Toc157947377)

[1.6 Dates 4](#_Toc157947378)

[1.7 Conclusions 4](#_Toc157947379)

[2 Sample details 5](#_Toc157947380)

[2.1 Sample description 5](#_Toc157947381)

[2.1.1 Sample source description 5](#_Toc157947382)

[2.1.2 Sample description 5](#_Toc157947383)

[2.2 Sample collection 5](#_Toc157947384)

[2.3 Sample storage 5](#_Toc157947385)

[2.4 Sample characteristics 5](#_Toc157947386)

[2.5 Sample dilution 5](#_Toc157947387)

[2.6 Sample staining 6](#_Toc157947388)

[2.7 Fluorescence reagents 7](#_Toc157947389)

[3 Flow cytometer 8](#_Toc157947390)

[3.1 Model and manufacturer 8](#_Toc157947391)

[3.2 Configuration and settings 8](#_Toc157947392)

[3.2.1 Flow rate and acquisition time 8](#_Toc157947393)

[3.2.2 Light sources 8](#_Toc157947394)

[3.2.3 Detectors 8](#_Toc157947395)

[3.2.4 Trigger detector and threshold 8](#_Toc157947396)

[4 Assay controls 9](#_Toc157947397)

[4.1 Buffer-only controls 9](#_Toc157947398)

[4.2 Buffer with reagents controls 9](#_Toc157947399)

[4.3 Unstained controls 9](#_Toc157947400)

[4.4 Isotype controls 9](#_Toc157947401)

[5 Data analyses 11](#_Toc157947402)

[5.1 Data sharing 11](#_Toc157947403)

[5.2 Compensation details 11](#_Toc157947404)

[5.3 Calibrations 11](#_Toc157947405)

[5.3.1 Flow rate 11](#_Toc157947406)

[5.3.2 Fluorescence calibration 11](#_Toc157947407)

[5.3.3 Light scattering calibration 12](#_Toc157947408)

[5.4 Gate description and boundaries 13](#_Toc157947409)

[6 References 15](#_Toc157947410)

# Experiment overview

## Contact details

### Experiment leader

| Name of organization | Medical University of Warsaw |
| --- | --- |
| Address | Ulica Żwirki i Wigury 61  02-091 Warsaw  Poland |
| Primary contact name | Dr. Aleksandra Gąsecka – van der Pol |
| Primary contact e-mail address | [aleksandra.gasecka@wum.edu.pl](mailto:aleksandra.gasecka@wum.edu.pl) |

### Flow cytometry execution

| Name of organization | Amsterdam University Medical Centers |
| --- | --- |
| Address | Meibergdreef 9  PO Box 22660  1100 DD  Amsterdam  The Netherlands |
| Contact name | Edwin van der Pol |
| Contact e-mail address | [e.vanderpol@amsterdamumc.nl](mailto:e.vanderpol@amsterdamumc.nl) |

## Purpose

The goal of this flow cytometry experiment is to determine the effect of transcatheter aortic valve implantation (TAVI) on plasma concentrations of five extracellular vesicles (EVs) subtypes, and to evaluate the predictive value of these EVs for major adverse cardiac and cerebrovascular events (MACCE).

## Keywords

Extracellular vesicles, flow cytometry, human blood plasma, aortic stenosis, prognosis, transcatheter aortic valve implantation

## Experiment variables

The experimental variable is whether TAVI affects the concentrations of different EVs subtypes and whether EVs can be used to predict post-TAVI MACCE during the median observation period of 10.3 months. In total, 140 patients who were qualified TAVI due to aortic stenosis based on the Heart Team decision were included in the study. There were no healthy controls.

## Experiment design and quality controls

All samples were measured using an autosampler, which facilitates subsequent measurements of samples in a 96-well plate. The entire study involved fifteen 96-well plates that were measured within 12 months. Each well plate contained a buffer-only control, reagents in buffer controls and isotype controls. Flow rate, fluorescence, and light scattering calibrations were performed daily.

## Dates

Samples were collected between November 2018 and June 2020 at (i) 1st Chair and Department of Cardiology, Medical University of Warsaw, Poland, (ii) Department of Cardiology, Nowa Sól Multidisciplinary Hospital, Poland and (iii) Department of Cardiac Surgery and Transplantology, Poznan University of Medical Sciences, Poland. Flow cytometry experiments were performed between December 2020 and December 2021 at Laboratory of Experimental Clinical Chemistry, Amsterdam UMC, Amsterdam, The Netherlands.

## Conclusions

Total EV concentration and concentration of EVs from leukocytes decreased after TAVI, compared to the measurement before TAVI. Increased pre-TAVI concentration of EVs exposing phosphatidylserine was an independent predictor of MACCE in multivariable analysis. Pre-TAVI concentration of PS-exposing EVs correlated with echocardiographic parameters of AS severity.

# Sample details

## Sample description

### Sample source description

The study population includes patients qualified for TAVI due to aortic stenosis based on the Heart Team decision. Exclusion criteria were transcatheter valve-in-valve implantation, chronic kidney disease (glomerular filtration rate < 30 mL/min), autoimmune diseases, active neoplastic disease, pregnancy and breast-feeding. All patients provided informed written consent.

### Sample description

Venous collected blood.

## Sample collection

All details of sample collection are provided in the main manuscript.

## Sample storage

All details of sample storage are provided in the main manuscript.

## Sample characteristics

Frozen human plasma samples are expected to contain the following particles: erythrocyte ghosts, EVs, lipoproteins, platelets, precipitated salt crystals, proteins, and complexes of the aforementioned particles.

## Sample dilution

As the concentration of particles in plasma differs >10^2^-fold between donors, samples require different dilutions to (1) avoid swarm detection and (2) detect a statistically significant number of events within a measurement time of a few minutes. The optimal dilution factor is the minimum dilution factor that is required to prevent swarm detection. For the flow cytometer and settings used, the optimal dilution factor should result in a count rate <1.1∙10^4^ events∙s^-1^ [4].

To find the dilution resulting in a count rate <1.1∙10^4^ events∙s^-1^, we diluted each sample 1,000‑fold in Dulbecco′s phosphate buffered saline (DPBS) and measured the total concentration of particles for 30 s without staining. For all experiments, filtered DPBS (Corning, US) was used. By diluting each sample 1,000-fold, all samples had a count rate <1.1∙10^4^ events∙s^-1^. Figure 1A shows a distribution of the measured total particle concentrations of all samples in the study. Taking into account the measured concentration and flow rate, we calculated the minimum dilution factor required before staining (section 2.6) to achieve a count rate <1.1∙10^4^ events∙s^-1^ after staining. The staining procedure adds an extra dilution factor of 11.1-fold to the overall dilution. To simplify the pipetting procedures, samples were divided into 6 categories of pre-staining dilution factors: 2-fold, 3-fold, 5-fold, 7-fold, 10-fold, 16-fold, 25-fold, 40-fold, 65-fold, 100-fold, 160-fold, 200-fold and 260-fold. Figure 1B shows a distribution of the applied pre-staining dilution factors of all samples in the study.


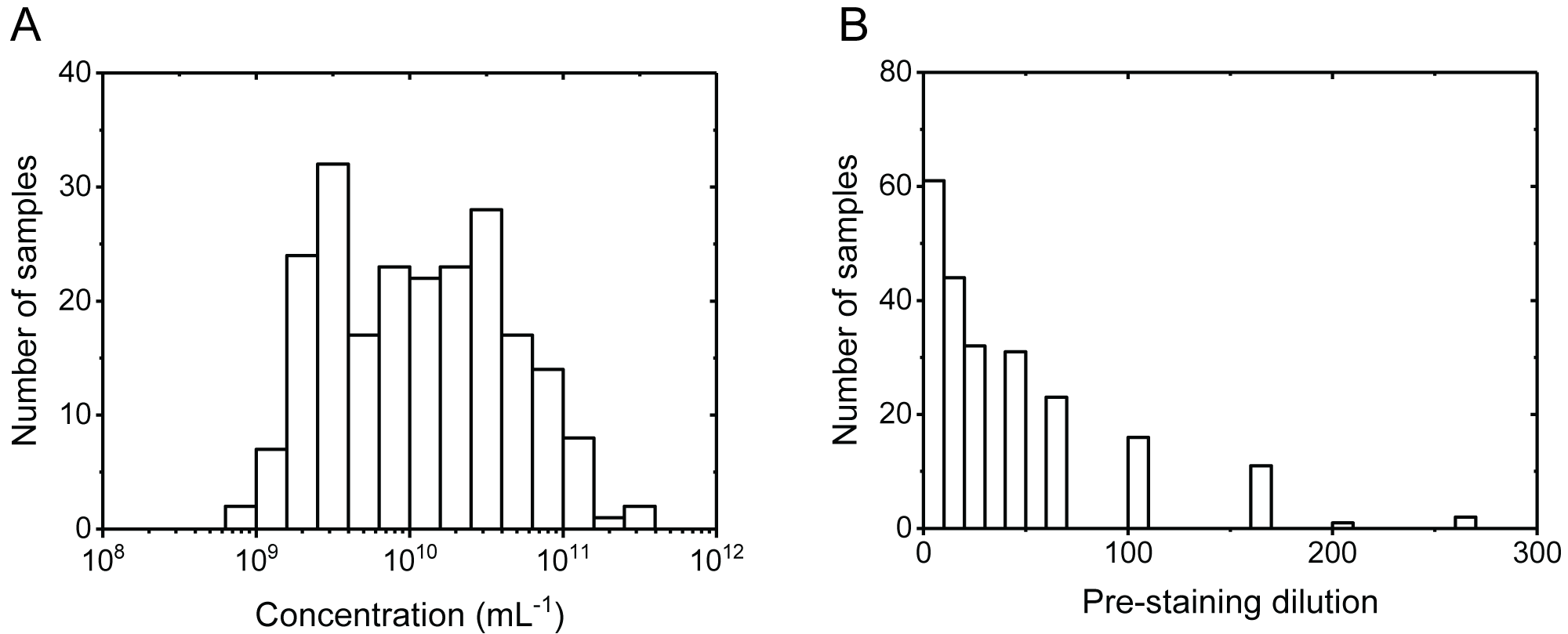


Figure 1: (A) Distribution of the total concentration of particles exceeding the trigger threshold for all samples in the study. (B) Distribution of the applied pre-staining dilution factors for all samples in the study.

## Sample staining

| **Characteristic**  **measured** | **Analyte** | **Analyte detector** | **Reporter** | **Isotype** | **Clone** | **Concentration (µg mL^-1^)** | **Manufacturer** | **Catalog number** | **Lot number** | **Dilution factor** |
| --- | --- | --- | --- | --- | --- | --- | --- | --- | --- | --- |
| Leukocyte common antigen | CD45 | Anti-human CD45  antibody | APC | IgG1 | 2D1 | 25 | BD Pharmingen™ | 340910 | 5040555 | 8x |
| Integrin | Human  CD61 | Anti-human CD61 antibody | APC | IgG1 | VI-PL2 | 50 | eBioscience™ | 17-0619-42 | 2062626 | 32x |
| Adhesion molecule | Human  CD62p | Anti-human CD62P antibody | PE | **IgG1** | CLB  Thromb/6 | 6.25 | Beckman Coulter | IM1759U | 37 | 16x |
| Adhesion molecule | CD146 | Anti-human CD146  antibody | PE | IgG1 | S-Endo 1 | 3.75 | Biocytex | 5050- PE100T | 173455 | 4x |
| Glycoprotein | CD235a | Anti-human CD235a  antibody | PE | IgG1 | JC159 | 100 | Dako | R7078 | 20056279 | 16x |
| Glycoprotein | Lactadherin | Lactadherin | FITC | n.a. | n.a. | 83 | Haematologic Technologies | Blac-FITC | GG1122 | 8x |
| Affinity for Fc receptor | Fc receptor | IgG1 | APC | n.a. | X40 | 200 | BD Pharmingen™ | 554681 | 7075605 | 100x |
|  | Fc receptor | IgG1 | PE | n.a. | IS5-21F5 | 50 | BD Pharmingen™ | 345816 | 7248665 | 50x |

Table 1 shows an overview of the antibodies that were used to stain EVs in plasma. Prior to staining, the antibodies were diluted in DPBS. For each antibody the optimal dilution factor was determined by titration (Table 1). To remove aggregates, diluted antibodies were centrifuged at 18,890 g for 5 min at 20 °C. The supernatant minus 10 μL of the starting volume was collected and used for staining. Each sample was (i) double stained with CD45-APC (allophycocyanin) and CD146-PE (phycoerythrin), (ii) double stained with CD61-APC and CD62p-PE, (iii) single stained with CD235a-PE, (iv) single stained with lactadherin-FITC (fluorescein isothiocyanate). To stain, 20 μL of pre-staining diluted (Figure 1B) plasma was incubated with 2.5 μL of each antibody or isotype controls and kept in the dark for 2 h at room temperature. After the incubation, samples were diluted in 200 μL DPBS to decrease background fluorescence from unbound reagents.

## Fluorescence reagents

| **Characteristic**  **measured** | **Analyte** | **Analyte detector** | **Reporter** | **Isotype** | **Clone** | **Concentration (µg mL^-1^)** | **Manufacturer** | **Catalog number** | **Lot number** | **Dilution factor** |
| --- | --- | --- | --- | --- | --- | --- | --- | --- | --- | --- |
| Leukocyte common antigen | CD45 | Anti-human CD45  antibody | APC | IgG1 | 2D1 | 25 | BD Pharmingen™ | 340910 | 5040555 | 8x |
| Integrin | Human  CD61 | Anti-human CD61 antibody | APC | IgG1 | VI-PL2 | 50 | eBioscience™ | 17-0619-42 | 2062626 | 32x |
| Adhesion molecule | Human  CD62p | Anti-human CD62P antibody | PE | **IgG1** | CLB  Thromb/6 | 6.25 | Beckman Coulter | IM1759U | 37 | 16x |
| Adhesion molecule | CD146 | Anti-human CD146  antibody | PE | IgG1 | S-Endo 1 | 3.75 | Biocytex | 5050- PE100T | 173455 | 4x |
| Glycoprotein | CD235a | Anti-human CD235a  antibody | PE | IgG1 | JC159 | 100 | Dako | R7078 | 20056279 | 16x |
| Glycoprotein | Lactadherin | Lactadherin | FITC | n.a. | n.a. | 83 | Haematologic Technologies | Blac-FITC | GG1122 | 8x |
| Affinity for Fc receptor | Fc receptor | IgG1 | APC | n.a. | X40 | 200 | BD Pharmingen™ | 554681 | 7075605 | 100x |
|  | Fc receptor | IgG1 | PE | n.a. | IS5-21F5 | 50 | BD Pharmingen™ | 345816 | 7248665 | 50x |

Table 1. Overview of staining reagents. Characteristics being measured, analyte, analyte detector, reporter, isotype, clone, concentration during staining, manufacturer, catalog number and lot number of used staining reagents. The concentration of staining reagents during measurements was 11.1-fold lower than the concentration during staining. APC: allophycocyanin; CD: cluster of differentiation; FITC: fluorescein isothiocyanate; IgG: immunoglobulin G; PE: phycoerythrin; BD: Becton Dickinson.

# Flow cytometer

## Model and manufacturer

A60-Micro, Apogee Flow Systems, Hemel Hempstead, UK. The flow cytometer has not been altered. All components are original and came with the flow cytometer.

## Configuration and settings

### Flow rate and acquisition time

The flow cytometer is equipped with a syringe pump with volumetric control. Samples were analysed for 120 s at a flow rate of 3.01 μL/min.

### Light sources

The flow cytometer has three lasers that illuminate a fixed-alignment cuvette flow cell. The laser powers were 100 mW, 150 mW and 150 mW for the 405-nm, 488-nm, and 638-nm laser, respectively.

### Detectors

Table 2 shows an overview of the detectors used in this study.

| Detector name | Detected property | Voltage (V) | Spectral filter bandwidth (nm) |
| --- | --- | --- | --- |
| 405-SALS | Forward scattered light | 470 |  |
| 405-LALS | Side scattered light | 375 |  |
| 488-Orange | PE fluorescence | 450 | 575/30 |
| 638-D Red | APC fluorescence | 480 | >650 |
| 488-Green | FITC fluorescence | 520 | 525/50 |

Table 2. Detector name, detected property, voltage and spectral filter bandwidth of the detectors used in this study. APC: allophycocyanin; FITC: fluorescein isothiocyanate; PE: phycoerythrin; SALS: small angle light scattering; LALS: large angle light scattering.

### Trigger detector and threshold

Based on the buffer-only control, a trigger threshold of 24 arbitrary units was applied to the side scattering detector. As (1) the A60-Micro applies the trigger threshold analogically, thus before digitalization of the signal, and (2) the arbitrary unit channel numbers differ between the data acquisition software and the flow cytometry datafiles, the trigger threshold was expressed in standard units by taking the mode of the effective side scattering cross section distribution and optical diameter distribution (section 5.3.3.1) of a plasma sample. Plasma samples have a size distribution with a peak far below the detection limit of the A60-Micro. The modes of the effective side scattering cross section distribution and optical diameter distribution therefore represent the trigger threshold. The trigger threshold is equivalent to an effective side scattering cross section of 10 nm^2^ and an optical diameter of 165 nm for EVs^[[1]](#footnote-1)^.

# Assay controls

Assay controls recommended by the MIFlowCyt-EV framework were performed to confirm that signals originate from EVs. Fluorescence-minus-one and single-stained controls were not performed due to thorough experience with the used antibody panels and because the emission spectra of the used fluorophores do not have spectral overlap. Procedural controls were not performed because no methods to isolate EVs were applied after staining. Serial dilution control were performed on the A60-Micro for six representative plasma samples and results were published [4]. Section 2.5 explains how swarm detection was prevented. Detergent treatment controls were not performed because they were not recommended at the time of the sample measurements.

## Buffer-only controls

Each 96-well plate contained at least 1 well with DPBS, which was measured with the same flow cytometer and acquisition settings as all other samples. The median count rate for all DPBS measurements was 32 events s^-1^, which is lower than the target count rate (7.0∙10^3^ events s^-1^) for events in stained plasma samples.

## Buffer with reagents controls

Each 96-wellplate contained a buffer with reagent control for each reagent (

| **Characteristic**  **measured** | **Analyte** | **Analyte detector** | **Reporter** | **Isotype** | **Clone** | **Concentration (µg mL^-1^)** | **Manufacturer** | **Catalog number** | **Lot number** | **Dilution factor** |
| --- | --- | --- | --- | --- | --- | --- | --- | --- | --- | --- |
| Leukocyte common antigen | CD45 | Anti-human CD45  antibody | APC | IgG1 | 2D1 | 25 | BD Pharmingen™ | 340910 | 5040555 | 8x |
| Integrin | Human  CD61 | Anti-human CD61 antibody | APC | IgG1 | VI-PL2 | 50 | eBioscience™ | 17-0619-42 | 2062626 | 32x |
| Adhesion molecule | Human  CD62p | Anti-human CD62P antibody | PE | **IgG1** | CLB  Thromb/6 | 6.25 | Beckman Coulter | IM1759U | 37 | 16x |
| Adhesion molecule | CD146 | Anti-human CD146  antibody | PE | IgG1 | S-Endo 1 | 3.75 | Biocytex | 5050- PE100T | 173455 | 4x |
| Glycoprotein | CD235a | Anti-human CD235a  antibody | PE | IgG1 | JC159 | 100 | Dako | R7078 | 20056279 | 16x |
| Glycoprotein | Lactadherin | Lactadherin | FITC | n.a. | n.a. | 83 | Haematologic Technologies | Blac-FITC | GG1122 | 8x |
| Affinity for Fc receptor | Fc receptor | IgG1 | APC | n.a. | X40 | 200 | BD Pharmingen™ | 554681 | 7075605 | 100x |
|  | Fc receptor | IgG1 | PE | n.a. | IS5-21F5 | 50 | BD Pharmingen™ | 345816 | 7248665 | 50x |

Table 1), which was

measured with the same flow cytometer and acquisition settings as all other samples. Table 3 shows a summary of the results of the buffer with reagents controls.

| Reagent | Mean number of fluorescence positive events in buffer  (120 s^-1^) | Mean number of fluorescence positive events in stained samples  (120s^-1^) | Mean number of fluorescence positive events in buffer / stained samples (-) |
| --- | --- | --- | --- |
| CD61-APC | 5 | 2444 | 0.002 |
| CD45-APC | 120 | 213 | 0.565 |
| CD62p-PE | 7 | 13 | 0.545 |
| CD146-PE | 1 | 6 | 0.182 |
| CD235a-PE | 34 | 1080 | 0.032 |
| Lac-FITC | 151 | 2783 | 0.054 |

Table 3. Results of the buffer with reagents controls. APC: allophycocyanin; CD: cluster of differentiation; FITC: fluorescein isothiocyanate; PE: phycoerythrin.

Data based on results of the buffer with reagents controls, CD45-APC and CD62p-PE positive events should be carefully interpreted.

## Unstained controls

Unstained controls were measured with a 1,000-fold dilution factor, which differs from the dilution factor with which the stained plasma samples were measured. Unstained controls were not considered in this analysi

## Isotype controls

Each 96-wellplate contained an arbitrary plasma sample stained with IgG1, which was

measured with the same flow cytometer and acquisition settings as all other samples. Table 4 shows a summary of the results of the isotype controls.

| Sample | Mean number of fluorescence positive events in isotype control (120 s^-1^) | Mean number of fluorescence positive events in stained samples  (120 s^-1^) | Mean number of fluorescence positive events in isotype control / stained samples (-) |
| --- | --- | --- | --- |
| CD61-APC | 3 | 2444 | 0.001 |
| CD45-APC | 3 | 213 | 0.014 |
| CD62p-PE | 8 | 13 | 0.602 |
| CD146-PE | 8 | 6 | 1.306 |
| CD235a-PE | 8 | 1080 | 0.007 |
| Lac-FITC | 36 | 2783 | 0.013 |

Table 4. Results of the isotype controls. APC: allophycocyanin; CD: cluster of differentiation; FITC: fluorescein isothiocyanate; PE: phycoerythrin.

Data based on results of the isotype controls, CD62p-PE and CD146-PE positive events should be carefully interpreted.

# Data analyses

To automatically determine optimal samples dilutions, apply calibrations, determine and apply gates, generate reports with scatter plots and generate data summaries, we developed and applied custom-build software (MATLAB R2020b, Mathworks, USA).

## Data sharing

Data are available upon request from the corresponding author (Aleksandra Gąsecka: [aleksandra.gasecka@wum.edu.pl](mailto:aleksandra.gasecka@wum.edu.pl))

## Compensation details

No compensation was applied because no fluorophore combinations with overlapping emission spectra were measured simultaneously.

## Calibrations

### Flow rate

At the start of each measurement day, we applied the automated quality control system ApoCal (#1524, Apogee Flow Systems), which checks whether the flow rate is within 20% of the adjusted flow rate of 3.01 µL∙min^-1^. For all days, the flow cytometer passed this quality control check.

### Fluorescence calibration

Calibration of the fluorescence detectors from arbitrary units (a.u.) to molecules of equivalent soluble fluorochrome (MESF) was accomplished using 2 µm Q-APC beads (2321-175, BD, USA), QuantiBright FITC beads (13734, Bangs Laboratories, USA), and SPHERO Easy Calibration Fluorescent Particles (AK01, Spherotech Inc., USA). Figure 2A and B show the 10-base logarithm of the MESF intensities for the MESF beads versus the 10-base logarithm of the measured median fluorescence intensity of each bead population. The data are fitted with a linear function. For each measured plasma sample, we added fluorescent intensities in MESF units to the flow cytometry data files using following equation:

| $\text{I(MESF)}={10}^{a\cdot\log_{10} \text{I(a.u.)}+b}$ | Equation 1 |
| --- | --- |

where I is the fluorescence intensity, and *a* and *b* are the slope and the intercept of the linear fits in Figure 2.


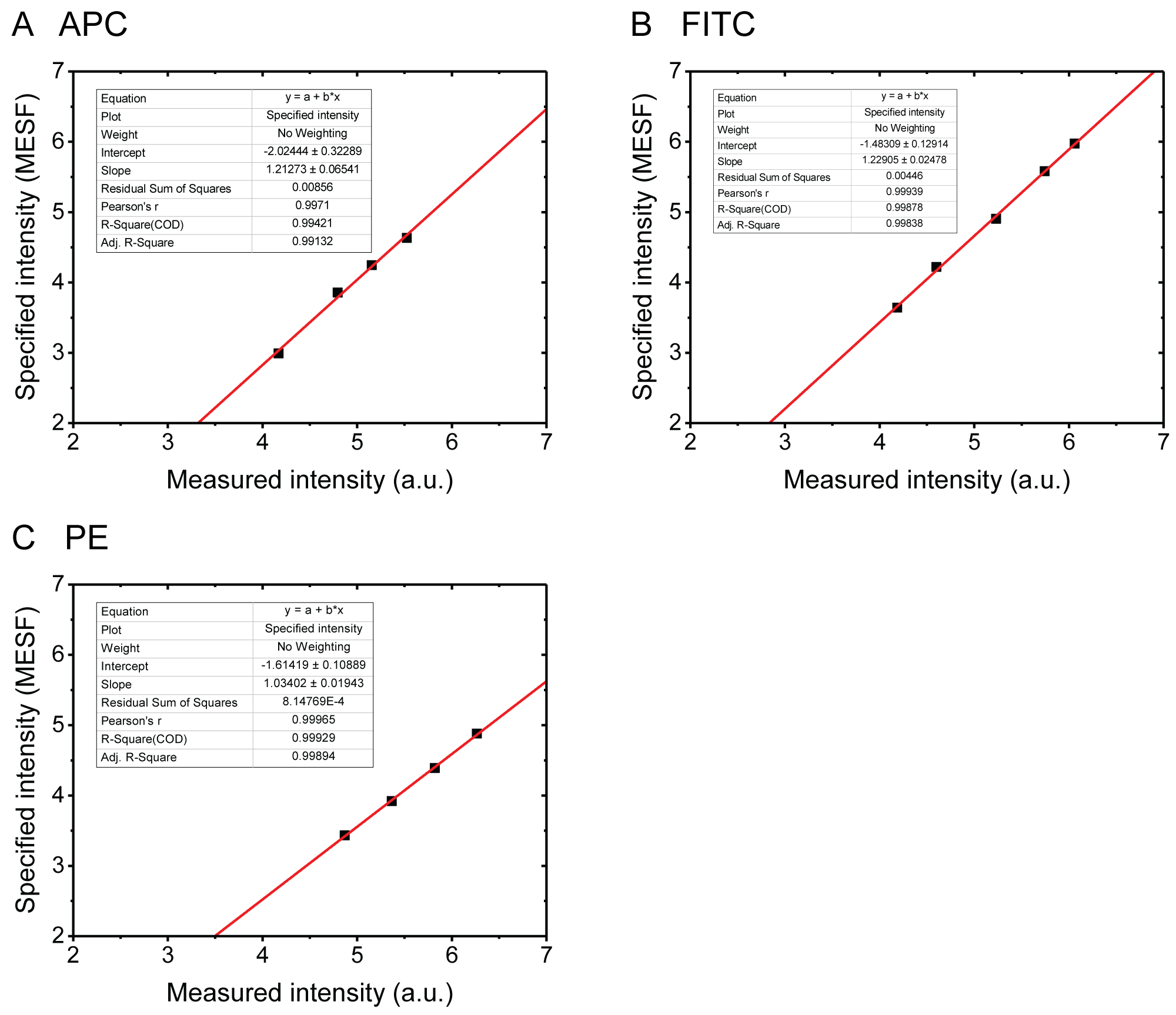


Figure 2. Calibration of the fluorescence detectors from arbitrary units (a.u.) to molecules of equivalent soluble fluorochrome (MESF). Logarithmic MESF versus logarithmic median fluorescence intensity for (A) allophycocyanin (APC), (B) fluorescein isothiocyanate (FITC), and (C) phycoerythrin (PE). Data (symbols) are fitted with a linear function (line).

### Light scattering calibration

#### Rosetta Calibration

Rosetta Calibration (v1.29, Exometry, The Netherlands) was used to relate the forward and side scattering intensities measured at a wavelength of 405 nm to the effective scattering cross sections^[[2]](#footnote-2)^ and optical diameter^[[3]](#footnote-3)^ of EVs. EVs are modelled as core-shell particles with a core refractive index of 1.38, a shell refractive index of 1.48, and a shell thickness of 6 nm. Figure 3 shows print screens of the light scatter calibrations.

A B


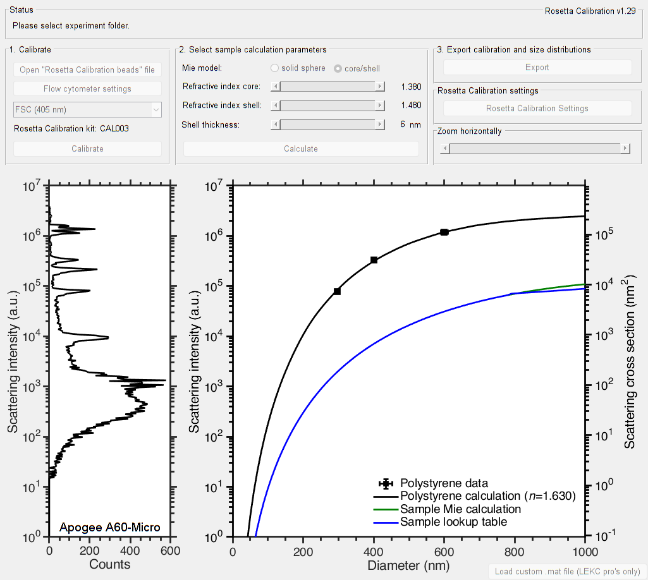

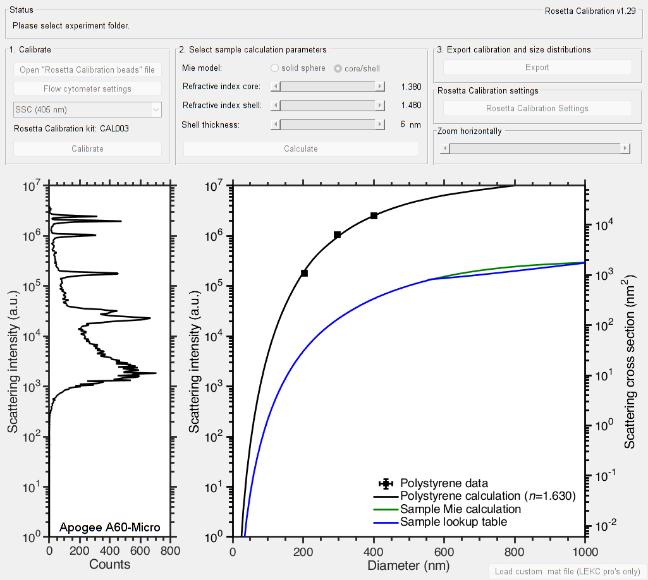


Figure 3. Forward scattering and side scattering calibration of the A60-Micro by Rosetta Calibration. To relate scatter to the diameter of EVs, EVs are modelled as core-shell particles with a core refractive index of 1.38, a shell refractive index of 1.48, and a shell thickness of 6 nm.

#### Flow Scatter Ratio (Flow-SR)

To determine the diameter and refractive index of particles and improve specificity by enabling label-free differentiation between EVs and lipoproteins, the flow scatter ratio (Flow-SR) was applied.

Flow-SR was performed as previously described [5,6]. Lookup tables were calculated for diameters ranging from 10 to 1000 nm, with step sizes of 1 nm, and refractive indices from 1.35 to 1.80 with step sizes of 0.001. The diameter and refractive index of each particle was added to the flow cytometry datafiles by custom-build software (MATLAB R2020b).

As Flow-SR requires accurate measurements of both forward scattering and side scattering, we applied Flow-SR only to particles with diameters >200 nm, as determined by Flow-SR, and fulfilling the condition:

| $\log_{10} \sigma_{SSC}>-0.7\cdot\log_{10} \sigma_{FSC}+3$ | Equation 2 |
| --- | --- |

where $\sigma_{SSC}$ is the side scattering cross section and $\sigma_{FSC}$ is the forward scattering cross section.

## Gate description and boundaries

The following gates have been applied to all flow cytometry data files by custom-build software (MATLAB R2020b):

1. During the second that an event was measured, the count rate was within 1000 counts per second from the median count rate of the entire measurement, and…
2. The diameter, as determined by Flow-SR, is between 200 nm and 800 nm, and…
3. the condition in Equation 2 is fulfilled, and…
   1. the refractive index, as determined by Flow-SR, is <1.42 to omit false positively labeled lipoproteins, and…
      1. the CD45-APC fluorescence >190 MESF, or…
      2. the CD62p-PE fluorescence >121 MESF, or…
      3. the CD61-APC fluorescence >190 MESF, or…
      4. the CD146-PE fluorescence >121 MESF, or…
      5. the CD235a-PE fluorescence >134 MESF, or…
      6. the lactaherin-FITC fluorescence > 550 MESF.

PDF files with scatter plots of all applied gates are available upon request (section 5.1). The fluorescence gates, which differentiate positively stained particles from background noise, were automatically determined with a publicly available MATLAB script using a tuning factor of 2 [7].

# References

[1] Théry C, Witwer KW, Aikawa E, Alcaraz MJ, Anderson JD, Andriantsitohaina R, Antoniou A, Arab T, Archer F, Atkin-Smith GK, others. Minimal information for studies of extracellular vesicles 2018 (MISEV2018): a position statement of the International Society for Extracellular Vesicles and update of the MISEV2014 guidelines. *J Extracell Vesicles* 2018; **7**: 1535750.

[2] Lee JA, Spidlen J, Boyce K, Cai J, Crosbie N, Dalphin M, Furlong J, Gasparetto M, Goldberg M, Goralczyk EM, others. MIFlowCyt: the minimum information about a Flow Cytometry Experiment. *Cytom Part A* Wiley Online Library; 2008; **73**: 926–30.

[3] Welsh JA, van der Pol E, Arkesteijn GJA, Bremer M, Brisson A, Coumans F, Dignat-George F, Duggan E, Ghiran I, Giebel B, Görgens A, Hendrix A, Lacroix R, Lannigan J, Libregts SFWM, Lozano-Andrés E, Morales-Kastresana A, Robert S, de Rond L, Tertel T, Tigges J, de Wever O, Yan X, Nieuwland R, Wauben MHM, Nolan JP, Jones JC. MIFlowCyt-EV: a framework for standardized reporting of extracellular vesicle flow cytometry experiments. *J Extracell Vesicles* 2020; **9**: 1713526.

[4] Buntsma NC, Shahsavari M, Gasecka A, Nieuwland R, van Leeuwen TG, van der Pol E. Preventing swarm detection in extracellular vesicle flow cytometry - a clinically applicable procedure. *Res Pract Thromb Haemost* Wiley; 2023; **7**: 100171.

[5] van der Pol E, de Rond L, Coumans FAW, Gool EL, Böing AN, Sturk A, Nieuwland R, van Leeuwen TG. Absolute sizing and label-free identification of extracellular vesicles by flow cytometry. *Nanomed Nanotechnol Biol Med* 2018; **14**: 801–10.

[6] de Rond L, Libregts SFWM, Rikkert LG, Hau CM, van der Pol E, Nieuwland R, van Leeuwen TG, Coumans FAW. Refractive index to evaluate staining specificity of extracellular vesicles by flow cytometry. *J Extracell Vesicles* 2019; **8**: 1643671.

[7] Gankema AAF, Li B, Nieuwland R, van der Pol E. Automated fluorescence gating and size determination reduce variation in measured concentration of extracellular vesicles by flow cytometry. *Cytom Part A* Wiley Online Library; 2022; **101**: 1049–56.

[8] Welsh JA, Arkesteijn GJA, Bremer M, Cimorelli M, Dignat-George F, Giebel B, Görgens A, Hendrix A, Kuiper M, Lacroix R, others. A compendium of single extracellular vesicle flow cytometry. *J Extracell Vesicles* Wiley Online Library; 2023; **12**: e12299.

1. EVs are modelled as core-shell particles with a core refractive index of 1.38, a shell refractive index of 1.48, and a shell thickness of 6 nm. [↑](#footnote-ref-1)
2. The effective scattering cross section is a hypothetical area of a particle that incoming light must impinge in order to be scattered towards the lens. The calibrated effective scattering cross section axis is independent of refractive index assumptions, but depends on the illumination wavelength and collection angles of the flow cytometer [8]. [↑](#footnote-ref-2)
3. The optical diameter equals the physical diameter of a particle when (1) the particle is spherical and (2) the particle has the same refractive index distribution as assumed in the model. [↑](#footnote-ref-3)
